# Menopause and bipolar disorder: Bridging research gaps and exploring postmenopause

**DOI:** 10.1111/bdi.13483

**Published:** 2024-08-04

**Authors:** Francesco Attanasio, Valentina Fazio, Carlotta Pira, Elena Manfredi, Lorenzo Fregna, Cristina Colombo

**Affiliations:** ^1^ Department of Clinical Neurosciences Vita‐Salute San Raffaele University Milan Italy; ^2^ Department of Human Neuroscience Sapienza University of Rome Rome Italy; ^3^ Mood Disorder Unit, Department of Clinical Neurosciences IRCCS San Raffaele Scientific Institute Milan Italy

## 1 BIPOLAR DISORDER AND MENOPAUSE: NAVIGATING THE CLINICAL DILEMMA

“Will menopause change my condition?” and “Do I need to change my medications when I enter menopause?” These are questions often asked with palpable concern by women diagnosed with bipolar disorder (BD) as they face the prospect of menopause. These inquiries reflect the significant clinical challenges encountered during this phase, marked by rapid changes in clinical conditions, an increase in depressive episodes, and symptom alterations that challenge the efficacy of previously successful treatments.^1^, ^2^, ^3^

Despite the universality of menopause and ongoing advances in psychiatric research, these issues remain critically relevant in clinical practice for psychiatrists managing BD, leaving professionals grappling for reliable answers to provide to their patients.

The current body of literature exploring the relationship between BD and menopause is notably sparse and fraught with methodological limitations.

A detailed analysis by Perich et al.^2^ points out numerous deficiencies that underline the urgent need for dedicated research to enhance our understanding of how BD and menopause interact:

1. Many studies lack a consistent definition of menopause, with the majority also lacking a standardized measure of menopause status, such as the stages of reproductive aging workshop (STRAW) + 10 criteria.^4^, ^5^
2. Several studies did not confirm BD diagnoses, and the literature predominantly lacks detailed information on symptom severity and relapse rates in patients with BD during menopause.
3. Most existing studies lack basic data of adequate size concerning women with BD during menopause.
4. The majority of existing studies did not utilize a prospective design, and when such designs were employed, the follow‐up periods were typically short. Additionally, large‐scale longitudinal studies are conspicuously absent.
5. Additional limitations include the lack of data on the course of the illness, such as the number of prior episodes and the presence of mixed mood or rapid cycling.

Despite these identified gaps, as evidenced by more recent reviews by Truong and Marsh^3^ and Aragno et al.,^1^ these problems are still largely overlooked, and the issue of BD and menopause remains largely unresolved. The continuous oversight in addressing these critical aspects indicates a significant area of need within psychiatric research and clinical practice.

## 2 POSTMENOPAUSE: AN UNEXPLORED CHAPTER IN BD RESEARCH

Expanding upon the critical research needs highlighted by Perich et al. there is a pressing need for focused investigation into postmenopause (PM), currently a major gap in our understanding and management of BD during menopause.

PM, according to the STRAW + 10 criteria,^4^, ^5^ particularly during phases +1c and +2, is characterized by hormonal stabilization marked by high levels of FSH and low levels of estradiol. This hormonal stability contrasts with the fluctuations observed during the menopausal transition (MT), making PM a distinct phase that requires independent study.

Given the significant research gaps outlined, we believe it is essential to focus on PM in a specific and independent manner in future studies. The following key reasons highlight the necessity of this targeted research approach:

1. Every woman will naturally progress to PM. With the worldwide life expectancy for women steadily increasing, more women will undergo PM than ever before.
2. To date, there are no exclusive studies focused on PM in the context of BD. The limited existing literature predominantly targets the MT, likely due to its significant hormonal and symptomatic fluctuations, despite its shorter duration.
3. The impact of PM on the course and clinical manifestations of BD has not been adequately studied. Existing literature only reports a general trend toward an increased frequency of mood disturbances, with a prevalence of depressive symptoms.^1^, ^2^, ^3^
4. PM is often mistakenly associated only with advanced age. However, PM typically begins about 2 years after the onset of menopause, occurring physiologically soon after the average age of 50, and can occur earlier due to pathological or iatrogenic conditions.^4^, ^5^ This highlights the necessity of studying PM independently from aging, as they likely have independent effects on the course of BD.
5. PM is unique in a woman's life because, unlike the fertile years and MT, which are characterized by significant hormonal fluctuations, PM offers a period of significant hormonal stability that is highly uniform among women.^4^ This stability presents a unique opportunity to systematically explore standardized therapeutic approaches that could significantly improve the management of BD during this phase.

## 3 FUTURE PERSPECTIVES AND CONCLUSION

The questions about how symptomatology and therapy for BD will change during menopause, raised at the beginning of this manuscript, remain unanswered but serve as crucial guides for future research.

The fact that PM has never been studied independently as a phase represents both a challenge and an opportunity for future optimization of interventions in BD.

It is now crucial to focus on understanding the impact of PM on BD. Clear definitions are needed, including the diagnosis of BD, its subtypes, and specifiers. Additionally, the specific phase of PM must be defined according to the STRAW + 10 criteria,^4^, ^5^ particularly focusing on phases +1c and +2, which are characterized by hormonal stabilization. Research should also concentrate on specific acute phases of BD, especially depressive phases which are more common during menopause. Longitudinal studies are necessary to evaluate the number and characteristics of relapses. Identifying the most effective acute phase treatments is essential, including assessing their effects on specific symptom complexes that may worsen during PM, such as somatic anxiety symptoms and insomnia. It is also vital to determine which strategies can maintain stability and when it is possible to reduce or discontinue maintenance medications.

At our Mood Disorder Unit at the IRCCS San Raffaele Hospital in Milan, Italy, we are actively contributing to this area by recruiting women diagnosed with BD specifically in PM. We encourage other research and care centers to undertake similar recruitment efforts, with the ultimate goal of drafting specific guidelines to aid psychiatrists in the delicate management of BD during PM. This has the potential to revolutionize the management of BD during this phase.

It is our hope that these recommendations will foster and support the efforts of the scientific and clinical community to better meet the needs of an increasing number of women living with BD during PM. By enhancing our understanding of specific pharmacological management during this phase, we can minimize trial‐and‐error approaches and reduce the burden of maintenance therapies. Streamlining treatment during PM not only improves the quality of life but also aligns with a broader strategy of personalized medicine, ensuring that treatments are both effective and better tolerated by patients at a stage when they may be less resilient to aggressive or unsuitable therapies.

## CONFLICT OF INTEREST STATEMENT

None of the authors report a conflict of interest.

## Data Availability

Data sharing not applicable to this article as no datasets were generated or analysed during the current study.
